# Novel Insight of CircRNAs in Cervical Cancer: Potential Biomarkers and Therapeutic Target

**DOI:** 10.3389/fmed.2022.759928

**Published:** 2022-06-23

**Authors:** Maomao Wu, Yanxun Han, Xiangfei Gong, Ke Wan, Yuchen Liu, Yi Zhou, Lizhi Zhang, Guozheng Tang, Hui Fang, Bangjie Chen, Fan Yang, Qing Zhao, Genbao Wang, Chenghao Zhanghuang, Yunling Zhang

**Affiliations:** ^1^Department of Pharmacy, Anhui Chest Hospital, Hefei, China; ^2^Department of Otolaryngology – Head and Neck Surgery, The First Affiliated Hospital of Anhui Medical University, Hefei, China; ^3^Wannan Medical College, Wuhu, China; ^4^School of Pharmacy, Anhui Medical University, Hefei, China; ^5^The First Clinical Medical College, Anhui Medical University, Hefei, China; ^6^Department of Pharmacy, Hangzhou Normal University Affiliated Hospital, Hangzhou, China; ^7^Department of Urology, Kunming Children’s Hospital, Kunming, China

**Keywords:** circRNA, cervical cancer, physiological functions, mechanism, HCC

## Abstract

Cervical cancer (CC) is a prominent cancer around the globe, with a high incidence, and fatality rate. Numerous recent investigations have shown that various non-coding RNAs are associated with the progression of CC. Circular RNAs, a novel class of non-coding RNAs, have a single chain covalent closed-loop structure and are involved in cell growth and other physiological processes. These dysregulated circRNAs seem to have environment-specific functions. They have been demonstrated in certain studies to have a dual involvement in oncogene production and tumor inhibition in different cell settings. Simultaneously, some evidence indicates that circRNAs are abnormally expressed in CC and contributes to its progression. Thus, the distinctive expression profile of circRNAs is associated with the diagnosis, prognosis, and treatment outcomes of CC. We summarized numerous CC-specific circles and their function in revealing the molecular processes of carcinogenesis and progression in CC in this review. Taken together, these data suggest that circRNA may be used as an early detection biomarker and potential therapeutic target in patients with CC.

## Introduction

Cervical cancer (CC) is the fourth most often diagnosed cancer in women and the fourth most frequently fatal cancer in women with substantial geographical variation in CC morbidity and mortality (1). Although preventive methods against CC such as cervix cancer screening and the human papillomavirus (HPV) vaccine are now widely used, the morbidity associated with CC remains quite high. Even more concerning are the mortality rates associated with CC following prognosis, despite the availability of effective treatments such as radical trachelectomy, pelvic lymph node dissection, and radiotherapy (2). Additionally, CC has the highest cancer rate among women in developing countries, accounting for approximately 87 percent of CC deaths (3). For instance, CC is the third most common type of gynecologic cancer in Iran (4) and is even more prevalent in India, where it is the second most common type (5). Additionally, previously identified risk factors for CC include smoking, oral contraceptive use for more than 5 years, HIV/AIDS diagnosis, and persistent infection with certain types of HPV (6). Despite the fact that advanced therapies have been implemented continuously over the last few years, the survival rate of CC remains difficult to improve. Numerous studies indicate the interaction between various oncogenes or tumor suppressor genes and CC, to gain a better understanding of the disease’s exact mechanism and to develop more effective treatments.

Non-coding RNAs (NcRNAs), once regarded as “junk DNA,” are categorized into microRNAs (miRNAs), small nucleolar RNAs (snoRNAs), long ncRNAs (lncRNAs), and circular RNAs (circRNAs). They could effectively provide feedback to a larger RNA communication network and ultimately regulate the fundamental protein effectors that have various cellular functions such as cell proliferation and differentiation (7). For example, many lncRNAs, dysregulated in prostate cancer (PC), are closely related to the tumorigenesis, metastasis, and prognosis of PC (8). Of note, circRNAs, emerging during RNA splicing, are the novel stars of the ncRNA world. Differing from linear RNAs terminated with 5’ caps and 3’ tail, circRNAs are circularized in a reaction termed “backsplicing,” whereby the spliceosome fuses a splice donor site in a downstream exon to a splice acceptor site in an upstream exon. The recent revelation of the widespread existence of circRNAs sheds an attractive light on the research of human diseases. Expression profile studies have found that circRNAs are developmentally regulated, tissue, and cell-type specific, and shared across eukaryotes. Besides, functional studies suggested that circRNAs were implicated with various diseases, including cancer. For instance, the recent studies demonstrated that circRNAs played the significant roles in diseases of cardiovascular system, such as cardiac fibrosis and heart failure (HF) (9). Meanwhile, several circRNAs such as hsa_circ_0018289 (10), circRNA-000284 (11), hsa_circ_0023404 (12), circRNA8924 (13), and circ_0067934 (14) have been proved to be involved in the progression of CC. CircRNA expression profiles revealed that hsa_circ_0018289 expression was increased in CC, implying that it promoted tumorigenesis (10). Specifically, the circRNAs mentioned above were all found to be upregulated in CC, playing an oncogenic role by regulating different pathways which would be discussed later. Nevertheless, much mechanism-relevant information about the roles of circRNAs in CC remains to be clarified before the full article is published.

Circular RNA is undoubtedly involved in the pathogenesis of CC, based on the previous research findings. Additional research in this area will significantly improve the diagnosis, prognosis, and treatment of CC. In this review, we discussed new perspectives of circRNAs in CC, including their roles in the tumorigenesis of CC integrating with the informed researches of miRNA and potential therapeutic functions. The purpose of this review is to summarize the emerging evidences and propose a new idea to explain how CC is coordinated by circRNAs.

## Overview of CircRNA

CircRNAs were initially discovered in RNA viruses, such as plant viroids as early as 1976, and were also found in eukaryotes as endogenous RNA splicing products in 1979 (15). Reflecting on their low abundance, they were first thought to be a result of splicing errors for several decades after the 1970s. However, with the development of RNA sequencing (RNA-seq) technologies and bioinformatics, the abundance and diversity of circRNAs were identified (16). For example, circRNA expression was coupled to upregulation of the linear host transcripts during neuronal differentiation in both cell line systems and primary neuron culture. The expressions of some circRNAs detected in extracellular body fluids such as saliva, blood, and urine (17) were even higher than those of their canonical linear transcripts of the same genes such as circHIPK3 (18).

**FIGURE 1:**
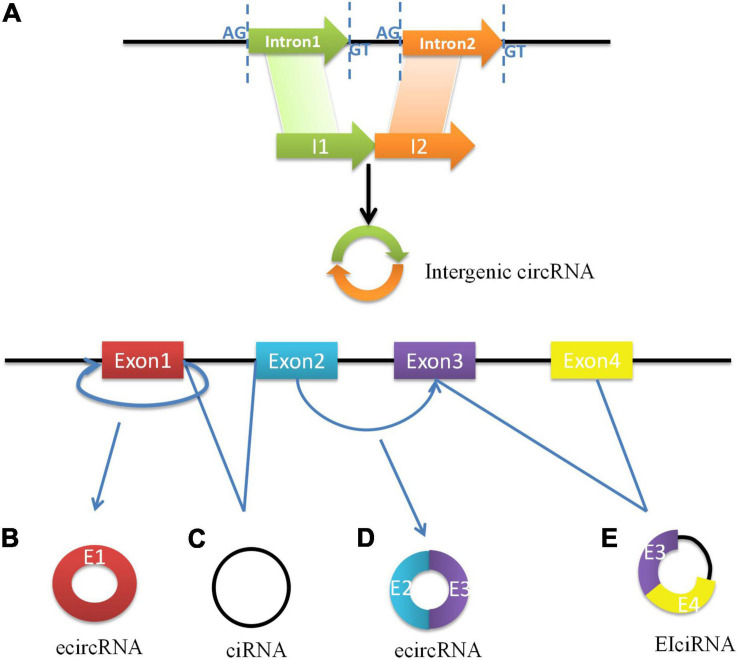
The biosynthesis of circRNAs. **(A)** Intergenic circRNAs consisted of introns are formed by covalent bonding between the splicing sites which are flanked by GT-AG signals. **(B)** ecircRNA is formed through backsplicing of the 5’ splice site (donor site) to a 3’ splice site (acceptor site) of exon 1. **(C)** The excised lariat intron can pair to produce ciRNA. **(D)** The intron between exon 2 and exon 3 is removed and the 5’ splice site of exon 3 is brought close to 3’ splice site of exon 2, which formed ecircRNA with exons more than one. **(E)** The intron’s escape from removal between exon 3 and exon 4 makes the formation of EIciRNA.

As a best-known family of RNA molecules, messenger RNA (mRNA) is transcribed by a single strand of template DNA and conveys genetic information from DNA to the ribosome which directs the protein synthesis. However, the primary transcripts designated to be mRNAs are called precursor mRNAs (pre-mRNAs) before modification for translation. Pre-mRNAs are spliced into linear molecules that alternately join the exons and retain the exon order (Alternative splicing). Higher eukaryotes can produce multiple mature RNAs and their respective protein products through alternative splicing (19). A few decades ago, it was shown that exon transcripts in pre-mRNAs might also be non-linearly reverse-spliced into circRNAs. Subsequent research established that circRNA is generated by the circulation of a single exon, multiple exons, exons, and introns (20). CircRNAs are classified as exon circRNAs (ecircRNAs), circular RNA from introns (ciRNAs), exon intron circRNAs (EIciRNAs), and intergenic circRNAs. Most ecircRNAs are mainly generated from back-spliced exons (16). The ecircRNAs predominantly remain in the cytoplasm. Moreover, lariat intron’s failure to debranch at the branch point site and the trimming of the lariat tail leading to the formation of circular intronic RNAs (ciRNAs) basically found in the nucleus (21). Exon–intron circRNAs are a distinct type of circRNA that is circularized with both exons and introns at the same time. Internal repeat sequences may play important roles in their generation, and they might be similar to ecircRNAs (22). Recently, Gao et al. identified intergenic circRNAs by circRNA identifier (CIRI), which may contain two intronic circRNA fragments flanked by GT-AG splicing signals acting as the splice donors or acceptors of the circular junction (23). The biosynthesis of circRNAs is shown in Figure 1.

**FIGURE 2:**
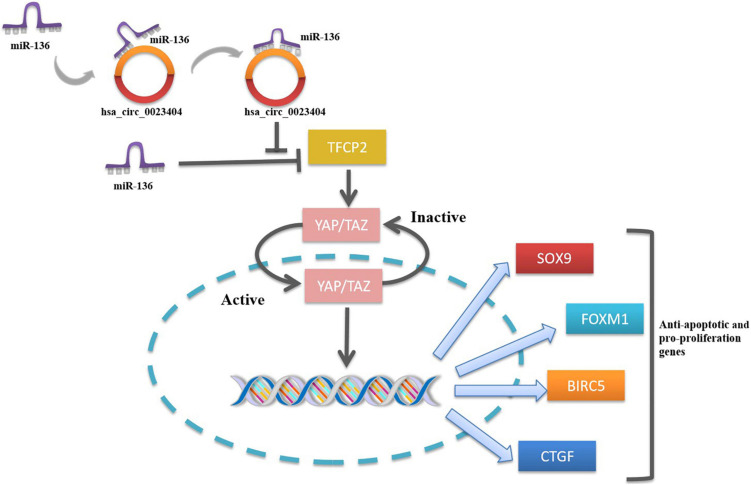
The hsa_circ_0023404 -miR-136-TFCP2-YAP signaling pathway axis.

CircRNAs play the important roles in physiological processes due to its conservation, abundance, and cell and tissue-specific expression. Of note, novel researches monitoring the participation of circRNAs in the origins of diseases indicated that circRNAs were indispensable in the progression of human diseases, especially in cancer. For example, Feng et al. discovered that circ-0000190 may impact myeloma cell proliferation, apoptosis, and cycle through the miR-767-5p/MAPK4 pathway (24). Additionally, another research shown that circ-ENO1 and its host gene ENO1 are increased in lung adenocarcinoma, promoting the glycolysis process and affecting tumor development and metastasis through the miR-22-3p/ENO1 axis (25). Furthermore, a recent study revealed that dysregulation of circRNA_100876 expression leads to a poor prognosis in esophageal squamous cell carcinoma (ESCC) by accelerating cell proliferation and metastasis (26). CircABCB10 could act as a tumor promoter in non-small cell lung cancer (NSCLC) by sponging miR-1252, providing the potential biomarkers and therapeutic targets for the management of NSCLC, etc. (27). To date, an array of circRNAs was found in different tissues and cells but we have only caught a glimpse of the whole complexity of the validated functions of circRNAs in diseases. Different types of circRNAs may perform different properties and exert their effect in varied ways. They can participate in the regulatory networks either as transcriptional regulators in nuclear or post-transcriptional regulators in cytoplasm (28). Nuclear circRNAs regulate RNA transcription by binding the cognate DNA locus or RNA polymerase II. Researchers found that circRNAs such as ecircRNAs could bind strongly to its cognate DNA locus, forming an RNA-DNA hybrid, or R-loop, resulting in transcriptional pausing (29). On the contrary, EIciRNAs display a positive regulatory role in transcription *via* specific RNA–RNA interaction with U1 snRNA (22). Cytoplasmic circRNAs primarily have function as miRNA sponges that inhibit miRNA expression and weaken the translation suppression on the corresponding target molecules (15). ecircRNAs in cytoplasm can act as “scaffolding” for RBPs by binding multiple RBPs (RNA-binding proteins), because they adopt the tertiary structures distinct from related linear molecules of the same sequence (30). Intriguingly, circRNAs can also serve as translation templates translating into protein but only in hepatitis delta virus hitherto (31). The functions of nuclear and cytoplasmic circRNAs are shown in Figure 2.

The universal cognition of circRNAs was turned from a rare mischief of epigenetics to budding stars with central regulatory roles in RNA metabolism. CircRNAs play a crucial role in different stages of biological processes, including cell development, proliferation, metastasis, fate decision, migration, and invasion. Amounting studies digging deeper into the mechanisms demonstrated that circRNAs can function as microRNA (miRNA) sponges, regulators of splicing and transcription, and modifiers of parental gene expression (32). Current studies also suggested that circRNAs had potential clinical values in human cancers, special focus on circRNAs in CC in this review.

## Overview of Cervical Cancer

Cervical cancer is a severe type of cancer in women around the world following the top three most common gynecologic malignancy, with 266,000 new death cases annually. Globally, there are 528,000 new cases of CC per year, 85% of which occur in low-income countries (33). Statistics showed that nearly 90% of CC deaths occurred in developing parts of the world in 2012 (34). CC is more prevalent in Asian young women and often manifests as irregular vaginal bleeding, lower abdominal discomfort, infertility, and abnormal vaginal secretion. Treatment options for recurring and advanced CC have dramatically improved over the last 5 years. However, the average overall survival time for advanced CC is 16.8 months, and the 5-year overall survival rate for all stages is only 68% (35), which has a significant influence and damage on patients’ quality of life and prognosis. CC is a major burden in Asia, which was still ranked the number one of gynecologic cancers in India—a large developing country with the population only second to China (36). CC often prevails among women who have HPV infections, lower socioeconomic predictors, smoking habits, high parity, long-term combination oral contraceptive (COC) use, an increased number of sexual partners, and early age at first intercourse. Particularly, HPV, detected in more than 99% of CC, is essential for the malignant transformation (37). CC was categorized into three types by the World Health Organization (WHO): squamous, glandular (adenocarcinoma), and other epithelial tumors including adenosquamous carcinoma, neuroendocrine tumors, and undifferentiated carcinoma (38). Different types feature different characteristics in pathology and pathophysiology.

The International Federation of Gynecology and Obstetrics—FIGO divided the CC into 4 stages as Stages I, II, III, and IV. Stage I carcinoma is strictly confined to the cervix whereas the other 3 stages metastasize beyond the cervix (39). Early CC is often asymptomatic, while locally advanced disease could cause symptoms that include abnormal vaginal bleeding (also after coitus), discharge, pelvic pain, and dyspareunia (38). For patients with CC, there are two types of metastasis: hematogenous metastasis and lymphatic metastasis and the later type poses a higher risk of death to the patients (40). Metastasis of CC is often the main cause of failure in tumor treatment and prognosis. Thus, it is necessary to investigate possible factors involving in the metastasis of CC. Accumulating discoveries showed that certain genes, RNA, and proteins played the vital roles in metastasis. For instance, E6/E7 was first found to target the tumor suppressor p53 and pRb, respectively, which is necessary for the maintenance of the malignant phenotype (41). Some signaling pathways have also been verified to involve in the carcinogenesis of CC such as Wnt signaling pathway (42). Zhang et al. have found that E6/E7-P53-POU2F1-CTHRC1 axis promotes CC metastasis by activating the Wnt/PCP signaling pathway (43). However, it is still difficult to elucidate the complexity of the underlying molecular mechanisms for CC metastasis. Hitherto, the prevailing treatments of CC include conization, hysterectomy, chemoradiation, and chemotherapy (44). Chemotherapeutic drugs are effective, but this treatment is considered only palliative and rarely cured. Patients with metastatic CC have a poor prognosis and their median survival is only 8–13 months (45). Accordingly, it is very urgent to investigate the molecular mechanisms during the proliferation, apoptosis, and invasion of CC, thus making groundbreaking progress in therapeutic strategies.

To avoid the tragedy of the poor prognosis outcomes, novel biomarkers with high sensitivity and specificity are desperately needed for the early detection of CC. New discoveries found that distinct non-coding RNAs were enriched in the exosomes of CC compared to the normal tissues, which inspired us to use them as convenient and non-invasive biomarkers for CC (46). With rapid development in the diagnostic and prognostic measures and treatment strategies, patients with suspicious CC will be detected at an early stage. Once confirmed, they can acquire favorable prognosis after immediate and proper surgeries in the near future rather than missing the best operation opportunities.

## Roles of CircRNA in Cervical Cancer

Accumulating evidences have indicated that circRNAs are involved in many diseases, especially cancers. The correlation of circRNA in the regulation of tumorigenesis, including genesis, differentiation, migration, and metastasis, has been explored. Some evidences have confirmed that some circRNAs play carcinogenic or antitumor roles in cancers such as gastric cancer (GC) and hepatocellular carcinoma. Recently, many reports have supported that circRNAs are also contributed to the tumorigenesis in CC. Increasing researches showed that a large number of circRNAs were differentially expressed in CC, indicating that these circRNAs might be singularly regulated and exert potential functions in the progression of CC (47). Thus, this review will focus on demonstrating the functional roles of circRNAs during the pathological process of CC. The expression of circRNAs in CC is listed in Table 1.

**TABLE 1:** The expression of circRNAs in CC.

circRNA	Genome location	Sample	Expression change	Function in CC	References (PMID)
Hsa_circ_0018289	chr10:46968580–46969453	CC tissue (94.3%,33/35)	+	Oncogenic role	29156822
CircRNA8924 (hsa_circ_0141539)	chr1:2072008388–207201024	CC tissues (96.97%,32/33)	+	Oncogenic role	30007986
CircRNA-000284		HeLa, CaSki, SiHa, C-33A, SW756	+	Oncogenic role	29511454
Hsa_circ_0023404	chr11:71668272–71671937	CC tissues (53)	+	Oncogenic role	29738762
Circ_0067934		CC tissues (61)	+	Oncogenic role	30362562

## The Oncogenic Roles of CircRNA

After looking into the profiles of aberrantly expressed circRNAs in CC tissues, hsa_circ_0018289 was identified as a potential functional circRNA from 6 randomly selected overexpressed candidate circRNAs in a recent study. Hsa_circ_0018289, located at chr10:46968580–46969453, has a spliced length of 348 nt. hsa_circ_0018289 was validated to markedly upregulate in both CC tissues and cells relative to the controls. The expression pattern of hsa_circ_0018289 in CC tissues was validated in 94.3% (33/35) CC tissues compared with their adjacent non-tumor tissues whereas the pattern in cells was validated in 5 CC cell lines (HeLa, CaSki, SiHa, HT-3, and C33A) relative to human epidermal cell (HaCaT). Furthermore, knockdown of hsa_circ_0018289 inhibited the proliferation of HeLa and SiHa cells measured by CCK-8 assays. Moreover, the result of transwell assay showed that the migration and invasion ability of HeLa and SiHa cells *in vitro* was suppressed by circ_0018289 silencing. Of note, hsa_circ_0018289 could also be crucial in tumor growth *in vivo* as xenograft model experiments showed that hsa_circ_0018289 knockdown significantly decreased the tumor volumes and weights compared to the control group. The neoplastic volume of the experiment group that was injected with HeLa cells stably transfected with si-hsa_circ_0018289 was measured about 54.5% the volume of the control group 3 weeks after injection. As for weight, the experiment group only had the weight below 50.0% of the control groups. All those statistics indicated that hsa_circ_0018289 can boost tumor growth in a great degree *in vivo* (10). In conclusion, the hsa_circ_0018289 may be an indispensable candidate to the progression of CC and provides a novel insight into circRNAs into the carcinogenesis of CC.

CircRNA-000284 is formed by “direct splicing” from the second exon (1,099 bp) of HIPK3 gene flanked on either side by long introns. Previous studies have showed that circHIPK3 significantly affected cell proliferation in different human cells (18). Recently, the role of circRNA-000284 in CC was investigated by a novel study. In contrast to normal cervical epithelial cells, circRNA-000284 was found to be significantly upregulated in all of the 5 CC cell lines (human CC cell lines HeLa, CaSki, SiHa, C-33A, and SW756). Furthermore, the expression level of the cell proliferation marker Ki-67 was lowered after the circRNA-000284 knockdown. The Ki-67 protein is present during all active phases of the cell cycle except G(0), which makes it an excellent marker for determining the so-called growth fraction of a given cell population thus a good cell proliferation biomarker especially in tumors (48). In this research, the application of this biomarker indicated that circRNA-000284 knockdown hindered the proliferation of HeLa cells. Moreover, HeLa and SiHa cells were arrested at the G0 phase after the knockdown of circRNA-000284, thus suppressing the proliferation and invasion of CC (11). In summary, those data illustrated that circRNA-000284 is crucial in the tumorigenesis of CC and may represent as a new molecular diagnosis and prognosis marker of CC.

Hsa_circ_0023404, located at chr11:71668272–71671937, is a 180 nt covalently linked circRNA, which has been found to play a pivot position in the poor prognosis of human lung cancer (49). Interestingly, consistent with the results of the study in the lung cancer, Zhang et al. first found the oncogenic role of hsa_circ_0023404 in CC. The RT-qPCR result showed that hsa_circ_0023404 was significantly upregulated in CC tissues compared to adjacent normal tissues, which was consistent with the upregulated expression of hsa_circ_0023404 in CC cell lines including SiHa, HeLa, CasKi, C-33a, and C4-1 cells compared to HCerEpiC cells. Furthermore, hsa_circ_0023404 silencing inhibited the proliferation, migration, and invasion of CC. hsa_circ_0023404 knockdown remarkably suppressed the malignant behaviors of SiHa and HeLa cells by preventing them from entering into S phase (12). Additionally, overexpression of hsa_circ_0023404 in patients with CC predicted a poor prognosis analyzed by Kaplan–Meier curve, indicating its positive modulating position in the progression of CC. Based on the above data, it is reasonable to speculate that hsa_circ_0023404 exerts an oncogenic role in CC and can be used as a novel biomarker for the prognosis of CC and provide a potential target for CC therapy.

CircRNA8924 (circBase ID: hsa_circ_0141539) is an exonic circRNA with 186 nt spliced length. It is located in chr1:2072008388–207201024 with designated gene name Clorf116. Recently, a novel study revealed that circRNA8924 plays a vital role in the malignancy of CC. Tumor size, FIGO stage and myometrial invasion were correlated with the expression level of circRNA8924. The expression of circRNA8924 was found to be significantly upregulated in 96.97% (32/33) CC tissues when compared to their adjacent normal tissues. What is more, circRNA8924 knockdown reduced the proliferation rate of SiHa and HeLa cells, whereas the overexpression lentivirus transfection showed the reversed result. Of note, circRNA8924 silencing increased the number of SiHa and HeLa cells blocked in G0/G1 phase and decreased that in S phase, further confirming circRNA8924’s oncogenic role in the tumorigenesis of CC. Intriguingly, CBX8 protein was significantly reduced after circRNA8924 knockdown lentivirus transfection. As the core component of the PRC1 complex, CBX8 is closely related to the histogenesis, invasion, metastasis, and prognosis of the tumor and serves as an initial factor in the progression of multifarious cancers (13). For instance, knockdown of CBX8 restrained the proliferation of colorectal cancer cells (50), and proliferation, invasion, and cloning ability of ESCC cells (KYSE2 and KYSE510) were also suppressed by CBX8 silencing (51). Above all, the correlation between circRNA8924 and CBX8 provided a novel insight into molecular treatment of CC although relevant studies are not adequate, and further researches are still needed. The targets of circRNAs are listed in Table 2.

**TABLE 2:** The targets of circRNAs in CC.

CircRNA	Target	Cell lines	References (PMID)
CircRNA8924 (hsa_circ_0141539)	CBX8	Siha and HeLa	30007986
CircRNA-000284	Snail-2	SiHa and HeLa	29511454
Hsa_circ_0023404	TFCP2	SiHa and HeLa	29738762
Circ_0067934	EIF3C	SiHa and HeLa	30362562

Song et al. demonstrated that HSA levels were lower in CC cells than in matched normal tissues, _ circRNA_ 101996 is abundantly expressed in CC cells and hsa_ circRNA_ 101996 may function as a sponge for mir-8075 and subsequently target TPX2 in CC cells using bioinformatics analysis. Simultaneously, as shown by cell biology method,_circRNA_101996 mediated upregulation of TPX2 by mir-8075 promotes to CC proliferation, migration, and invasion. As a result, its level of expression is strongly correlated with TNM stage, tumor size, lymph node metastases, and prognosis in patients with CC (52). This bioinformatic analysis approach contributes to our understanding of the circRNA’s function in CC and lays the groundwork for future research on the circRNA molecular regulatory network’s role in CC. Cai et al. demonstrated that HSA may influence the expression of mouse bisection 4 (MDM4) through altering mir-150-5p and, ultimately, the expression of the p53 gene using western blot and immunohistochemistry_ circ_ 0000263. They discovered that hsa_circ_0000263 expression was considerably increased in CC cells. Additionally, HSA was knocked out that_circ_0000263 may decrease CC cell proliferation and migration (53).

Additionally, Chen et al. used RT-qPCR to identify circRNA in CC cells and tissue samples_ 0000285, as well as conducting associated functional investigations. They determined the presence of CIRC using RT-qPCR or western blotting. CeRNA network members 0000285/mir-197-3p/ELK1 may limit cell viability and colony formation, inhibit the G0/G1 phase of the cell cycle, trigger apoptosis and autophagy in CC cells, and control CC progression. Additionally, they discovered that circRNA in CC samples_ 0000285 had a considerably greater level of expression than the equivalent normal tissues. After circRNA_ 0000285 was removed, downstream Fus expression was considerably decreased, showing that circRNA_ 0000285 may enhance CC proliferation and metastasis by upregulating Fus and therefore give prospective therapeutic targets for CC research (54).

Further study focused on a circRNA, circ_0067934, the upregulation of which promotes tumor development in hepatocellular carcinoma (14) and lung cancer (55). Therefore, probably, hypothesis serves that circ_0067934 may also perform as a pivot in the progression of CC. Recently, a study that investigates the role of circ_0067934 in CC corresponds with the assumption. Circ_0067934 expression was first found to be upregulated in CC tissues compared to adjacent normal tissues by RT-qPCR. Additionally, the expression pattern was consistent with further analysis using 19 pairs of CC tissues and matched normal tissues. Moreover, circ_0067934 promotes tumor growth *in vivo*, and circ_0067934 knockdown significantly inhibited tumor growth in xenograft nude mouse model. Specially, CC tissues with lymph node metastasis showed upregulated expression of circ_0067934 than tissues without metastasis. Furthermore, western blot results illustrated that circ_0067934 was implicated in epithelial mesenchymal transition (EMT), which is a major factor for CC metastasis (14). As a crucial biological process that provides tumor cells with the ability to escape to distant regions, EMT was contributed to the metastasis of many types of cancer such as breast cancer (56) and colorectal cancer (57). Taken together, circ_0067934 expression was positively correlated in CC progression by promoting tumor growth and lymph node metastasis and may be a brand-new biomarker for the patients with advanced stage of CC.

In conclusion, the circRNAs studied thus far in CC are upregulated, play a carcinogenic role in the development of CC, and are expected to serve as a new biomarker for the disease. However, the field still remains in its infancy. Intensive further studies are needed to explore more circRNAs in CC. It is reasonable to speculate that some circRNAs playing roles as tumor suppressors would be found in CC increasing diversity to the circRNA world of CC in the future.

## Regulation Roles of CircRNA in Cervical Cancer

CircRNAs are typically expressed non-coding RNAs, whereas their biogenesis and chief functions are not well understood. A couple of potential functions of circRNAs have been increasingly discovered during the lengthy biological evolution. Certain studies have discovered that circular RNA containing a microRNA response element (MRE) may act as a competitive endogenous RNA for miRNA (ceRNAs). They, such as sponges, compete for miRNA binding sites, thereby impairing miRNA activity (58). Observation into the structure of circRNAs further explained the predominant characteristic of circRNAs as ceRNAs for abundant MREs are found in the closed-loop structure of circRNAs. For example, circular RNA sponge for miR-7 (CiRS-7) contains more than 70 selectively conserved miRNA target sites and is highly and widely associated with Argonaute (AGO) proteins in a miR-7-dependent manner (59). Moreover, circHIPK3 was found to be able to sponge 9 different kinds of miRNAs (18). Furthermore, multiple circRNAs have been found to sponge different kinds of miRNA with the function of regulating the expression of parental genes. For example, circMTO1 sponges for oncogenic miR-9 (60), circABCB10 is the sponge of miR-1252 (27), and circ-UBAP2 (hsa_circ_0001846) sponges for miRNA-661 (61). miRNAs can lead to translational repression and target mRNA degradation after miRNA is loaded into the RNA-induced silencing complex (RISC) where it directs the complex to target mRNAs (62). Therefore, figuring out the behavior of circRNAs functioning as miRNA sponges in different cells and tissues has been a focused point once upon a time.

Hu et al. found that circ_0067934 binds to miR545 in SiHa and Hela cells as a sponge and overexpressed CIRC_ 0067934 downregulated mir-545 levels in CC tissues (14). MiR-545 overexpression was found to play the tumor suppressive roles in CC by significantly suppressing the proliferation, reducing the colony number, and attenuating migration and invasion. Furthermore, human eukaryotic translation initiation factor 3 (EIF3C) was validated to be the target of miR-545 by RT-qPCR and western blot analysis, which indicated that there was a negative correlation between miR-545 and EIF3C expression in CC tissues. EIF3C, a highly complex multiprotein assembly, has multiple functions in translation and its misregulation was implicated in oncogenesis and the maintenance of a cancerous state (63). Studies have found that the overexpression of EIF3C gene has a positive effect on colon cancer cell survival and progression which can be suppressed by lentivirus-mediated infection of EIF3C siRNA. Therefore, EIF3C silencing could be considered as a novel therapeutic tool for colon cancer treatment (64). Moreover, further study has found that restoration of EIF3C reversed the effects of circ_0067934 knockdown, proving that circ_0067934 could interact with miR545 to inhibit its expressing level in CC. Taken together, these results demonstrated that circ_0067934 facilitates CC progression through inhibiting miR545 and upregulating EIF3C expression. Both miR545 and EIF3C genes can be gene therapy targets for CC. In further intensive study, circRNA-miRNA-mRNA pathways were constructed for investigating the effect of the crucial regulation pathways in CC, following the composition molecule with hsa_circ_0023404-miR-136-TFCP2, circRNA8924-miR-518d-5p/519-5p-CBX8, and circRNA-000284-miR-506-Snail-2, which were progressively involved in certain pathophysiologic processes when individually analyzed. In conclusion, the investigation of the significant circRNA-miRNA-mRNA pathway would shed new light on the pathogenesis of CC and identify potential therapeutic targets for patients with CC. Furthermore, it will establish a mature platform for future research on additional diseases. CircRNAs function as microRNA sponges in CC are listed in Table 3.

**TABLE 3:** CircRNAs function as microRNA sponges in CC.

circRNA	MicroRNA sponged	Cells	References (PMID)
Hsa_circ_0018289	miR-497	HeLa cells	29156822
CircRNA8924 (hsa_circ_0141539)	miR-518d-5p/519-5p	CC cells, SiHa, and Hela cells	30007986
CircRNA-000284	miR-506	HeLa, CaSki, SiHa, C-33A, and SW756 cells	29511454
Hsa_circ_0023404	miR-136	CC cells, SiHa, and Hela cells	29738762
Circ_0067934	miR-545	CC cells, SiHa, and Hela cells	30362562

**FIGURE 3:**
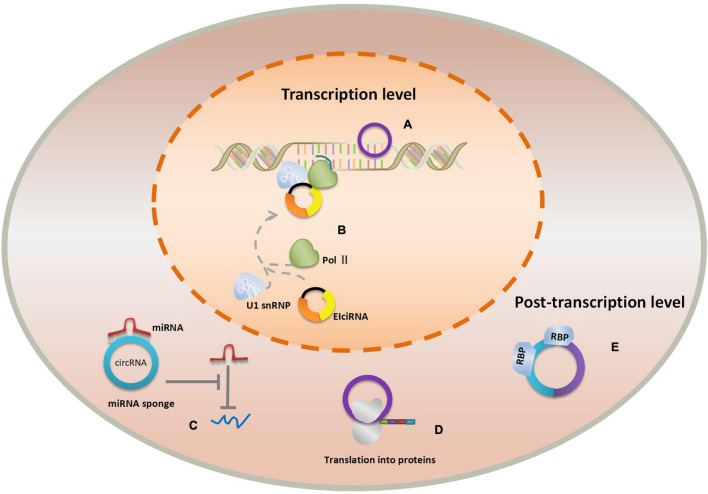
The functions of nuclear and cytoplasmic circRNAs. **(A)** ecircRNA’s effect of transcriptional pausing. **(B)** EIciRNA binds with Pol II and U1 snRNP to promote transcription. **(C)** circRNA in cytoplasm serves as miRNA sponge. **(D)** circRNA presents as translation template to produce protein. **(E)** Cytoplasmic ecircRNA acts as “scaffolding” for RBPs.

Of note, a novel study focused on the hsa_circ_0023404 -miR-136-TFCP2-YAP pathway axis in CC provided a more in-depth understanding of the possible pathological mechanism of CC. Zhang et al. for the first time demonstrated that hsa_circ_0023404 acted as a sponge of miR-136 and miR-136 targeted TFCP2, which is an activator of YAP signaling pathway. Yes-associated protein (YAP) is an important candidate in the YAP signaling pathway. When YAP was activated, they were free to translocate into the nucleus to promote cell proliferation. Nuclear YAP activates or suppresses transcription factors that regulate target genes with functions of cell proliferation, tissue growth, control of organ size and shape, or metastasis (65). Activating the YAP signaling pathway in CC *via* TFCP2 can significantly increase the expression of its target genes (Sox9, FoxM1, BIRC5, and CTGF), thereby promoting the proliferation, migration, and invasion of CC (12). By virtue of their interactions with miRNA, circRNAs play the key roles in regulating cancer progression and may involve in a variety of signaling pathways in cancers such as mitogen-activated protein kinase (MAPK)/extracellular signal-regulated kinase (ERK1/2), phosphatidylinositol 3-kinase (PI3K)/protein kinase B (AKT) intracellular signaling pathway, and Wnt/β-catenin pathway (20). Studies looking into the signal pathways regulated by circRNAs contribute to understanding the molecular regulatory mechanisms of the tumorigenesis. Therefore, further relevant studies are of great significance in the understanding of mechanism of circRNAs in CC. The hsa_circ_0023404 -miR-136-TFCP2-YAP signaling pathway axis is shown in Figure 3.

## Potential Clinical Application of CircRNAs in Cervical Cancer—The Biomarker Role

With ongoing advances and prevalence in the diagnosis approaches of CC, suspicious patients are more accessible to the detection of CC. However, the prognosis of patients with CC remains poor, with a 5-year overall survival of less than 30% in most countries (11). Regional or distant metastasis of tumor cells is the major reason behind the poor efficacy in treatment of CC. Thereby, early diagnosis is an important prerequisite for effective treatment of CC. Traditionally, the preventive examination of vagina and cervix smear, Pap test, and the HPV DNA test are the remarkable tools in the investigation of asymptomatic women and prognosis in the follow-up of women after the treatment of pre-invasive CC according to the American Cancer Association guidelines. Nonetheless, women with positive HPV test may have negative cytology (39), and the sensitivities of the Pap test can also be suboptimal (66). Therefore, it is imperative to discover more competent biomarkers to increase the diagnosis and prognosis accuracy of CC, thus improving the therapeutic effect on patients with CC. Indeed, the potential clinic values of circRNAs in CC have become the burning forefront among scientific researchers recently.

Wang et al. discovered that over 80,000 circRNAs were expressed in CCs and normal tissues, with approximately 25,000 being expressed in several ways. Notably, many of the circRNAs discovered by this microarray may be validated using RT-qPCR or RNA-seq. However, over 18,000 circRNAs can be successfully found in cell-free plasma samples, and the expression of around 2,700 circRNAs changes after tumor removal (67). This study results supported substantial evidence for circRNA expression in CC. Huang et al. identified a group of circRNAs in CC and matched normal tissues using RNA-seq data. They discovered that differentially expressed (DE) circRNAs may differentiate cancer from normal tissues, implying that the expression profile of circRNAs in CC is akin to that of normal tissues. There are notable distinctions (68). As discussed above, the aberrantly expressed circRNAs in CC were all significantly increased and promoted the progression of CC. Specially, overexpressed hsa_circ_0018289 and circ_0067934 were verified to promote tumor growth *in vivo* by the xenograft model experiments (10). Moreover, elevated expression of circRNA8924 (13) and circ_0067934 was found to be associated with the FIGO stage of CC (14). Upregulated circRNA8924 and circ_0067934 were also correlated with myometrial invasion and worse overall survival evaluated by Kaplan–Meier curve analysis, respectively, thereby acting as a promising prognostic biomarker of CC. Besides, hsa_circ_0023404 might also be a prognostic biomarker for patients with CC as the results of Kaplan–Meier curve analysis showed that overexpression of hsa_circ_0023404 in patients with CC predicted a poor prognosis (12). At the time, biomarkers circFoxO3a and circEIF4G2 associated with CC prognosis have been identified in CC tissues. Tang et al. used RT-qPCR to determine the level of circFoxO3a expression in the serum of patients with squamous CC and discovered that patients with squamous CC and low serum circFoxO3a expression had a poor prognosis in terms of overall survival and recurrence-free survival. Unfavorable prognostic indicators identified during this time frame may be employed as the non-invasive prognostic biomarkers (69). Mao et al. employed RT-qPCR to detect circEIF4G2 expression and discovered that its increased expression is strongly associated with tumor growth and lymph node metastasis. The researchers did a related Kaplan–Meier curve analysis and analyzed CC samples to see whether the expression of circEIF4G2 may be utilized as a prognostic factor for patients with CC. It found that increasing expression of circEIF4G2 was associated with reduced expression of circEIF4G2 in CC samples. The findings indicated that the greater the amount of circEIF4G2 expression in patients with CC, the poorer their survival rate. Thus, higher expression of circEIF4G2 in CC is strongly associated with a bad prognosis for patients with CC (70). Hence, it is rational to predict that those circRNAs can function as the biomarkers for CC and the field warrants further clinical verification.

Previous reports have already validated the enormous clinical values of certain circRNAs as the potent biomarkers for cancers. For instance, the value of hsa_circ_0001649 as a biomarker in HCC was examined by the receiver operating characteristic (ROC) curve, and the Cybulski area under the ROC curve (AUC) was 0.63 (71). In GC, hsa_circ_0000745 in plasma displayed excellent property of a diagnostic biomarker, which was evaluated by ROC curve and quantified by AUC being 0.683. Moreover, when combined with carcinoembryonic antigen (CEA), hsa_circ_0000745 had a better performance with an increased AUC of 0.775 (72). Taken together, all those cases above collaboratively confirmed that circRNAs can be the efficient biomarkers in the diagnosis and prognosis of cancers with higher sensitivity and specificity whether solely or integrated with other markers. Therefore, with further clinic study into the biomarker role of circRNAs, whether solely or combined with other makers, a circRNA will ultimately be verified as the excellent diagnostic or prognostic biomarkers of CC with high AUC.

Due to their endogenous regulatory functions, closed circular structure, high stability, involvement in cancer development, and progression, and differential expression in cancer tissues, circRNAs have the potential to serve as the diagnostic and prognostic biomarkers. Additionally, the dense circRNA found in various body fluids (such as blood, urine, or saliva) makes non-invasive detection of tumors possible, which is unmatched by traditional detection methods. Summarizing the observations above, we could find that circRNAs in different profiles such as hsa_circ_0018289, circRNA8924, circ_0067934, and hsa_circ_0023404 can be the promising biomarkers of CC for the earliest diagnosis and prognosis. Besides, Yi et al. discovered that five circulating RNAs (hsa_circRNA 000596, hsa_circRNA 104315, hsa_circRNA 400068, hsa_circRNA 101958, and hsa_circRNA 103519) may compete for endogenous RNA by forming a regulatory circular microRNA. These circular RNAs are involved in the regulation of mRNA rs5030743 and rs1130609 or other comparable SNPs used in the treatment of CC with targeted chemotherapeutic medicines (73). Additionally, recent gene sequencing studies revealed that CIRC expression was much greater in cervical tissue than in surrounding tissues. *In vitro*, CIRC expression was decreased that_ 0104541 inhibits the migration and invasion of CC cells considerably and may be employed as a therapeutic agent for CC. The disease’s potential biomarkers provide an exciting therapeutic opportunity for disease-directed treatment. Circular RNA is predicted to become a novel target for the treatment of CC as a result of these studies (74).

All of those progresses may be helpful to cure the patients with CC with more and more researches on this field in the future, and the veil covering the clinical values of circRNAs in CC will surely be lifted.

## Future Expectation

The relationship between miRNAs and circRNAs deserves to be a significant part of epigenetics. Thus, additional experiments on CC and other cancer types should be conducted to discover the individual or cooperative functions of the two non-coding RNAs before the coming full article.

Currently, there is a growing interest in developing nano-carriers used for co-delivery of small molecules and genes to treat cancer. Gene-based therapies using siRNA/miRNA have been suggested as new treatment methods to improve the current regimen (75). Therefore, recent studies verified that the co-delivery of IL17RB siRNA and DOX by nanoparticles can be considered as an effective system for the treatment of breast cancer resulting in a significant silencing of NF-κB and Bcl-2 relative gene expression, apoptosis induction, and migration inhibition in breast cancer cells (76). Moreover, miR-542-3p appended SRF/ATRA-loaded solid lipid nanoparticle was demonstrated for its therapeutic efficacy against GCs (77). As for CC, recently, poly (DL-lactide-co-glycolide) nanoparticles loaded with gold-ursolic acid were administered to CC cells to find effective treatments for CC inhibition due to the properties of ursolic acid in altering cellular processes and the easier absorbance of nanoparticles (78). Therefore, it may be reasonable to conclude that the co-delivery system *via* nanoparticles can be generalized to a broad spectrum of siRNAs, miRNAs, or circRNAs in the therapy of CC. This filed is worthy of wide attention.

The recent advent of genome-editing technologies has enabled a new paradigm in which the sequence of the human genome can be precisely manipulated to achieve a therapeutic effect. This includes the correction of mutations that cause disease, the addition of therapeutic genes to specific sites in the genome, and the removal of deleterious genes or genome sequences (51). Recent reports have provided proof of concept using CRISPR/Cas9 to successfully repair or inactivate mutations in animal models of monogenic human diseases. For example, CRISPR/Cas9 can be potentially applied in the treatment of monogenic lung diseases at a time when this technology is evolving rapidly (79). In recent studies of CC therapies, gene therapy was also applied and investigated. Tumor necrosis factor-related apoptosis-inducing ligand (TRAIL) and/or endostatin genes were delivered by nanoparticles to treat CC and were verified to offer considerable potential as an ideal candidate for cancer gene delivery therapy *in vivo* (80). From the researches about circRNAs discussed above, circRNAs played the important roles in the tumorigenesis of CC, and thus, gene therapy targeted on gene locations of circRNAs may be a promising therapy for CC. However, this is only a hypothesis based on the present studies, and further exploration is needed to probe into this new field.

According to the recent study, exosome, 30–100 nm in size, is a type of extracellular vesicles (EVs) composed of a lipid bilayer and well delimited round morphology. They participate in intercellular material transport and communication between nearby and distant cells (81). Liu et al. found that exosomes were abundant in the cervicovaginal lavage specimens of women with CC (82). With more deepening research of exosomes, lncRNAs are verified to be rich in exosomes released from CC cells. Expression of HOTAIR, MALAT1, and MEG3 was predominantly observed in CC-derived exosomes in cervicovaginal lavage samples (46). Moreover, Zhou et al. found that miR-221-3p was characteristically enriched in and transferred by CSCC-secreted exosomes into human lymphatic endothelial cells (HLECs) to promote HLEC migration and tube formation *in vitro* and facilitate lymphangiogenesis and LN metastasis *in vivo* (25). As for circRNAs, Li et al. first found that circRNAs were abundant in the exosomes. In addition, such abundance of circRNAs in the exosomes was much enlarged when compared to the producer cells (83). Sometimes, circRNAs were highly abundant in exosomes than cells. For example, Lasda and Parker found that EV circRNAs were better than the corresponding EV linear PCR (conventionally spliced RNAs originating from the same gene) when compared to the same ratio in cells of all three different cell lines. It was indicated that cells clear circRNAs by EVs and excreted circRNAs could contribute to cell communication with each other (84). Furthermore, circRNAs packaged into EVs played the important roles in the communication between cells in cancer in some cases. For example, circRNAs in plasma exosomes had specific expression features in GC, and exosome-delivered circRNAs were involved in WAT browning by activating PRDM16 and suppressing miR-133 in patients with GC (85). Thus, it is reasonable to speculate that extracellular circRNAs delivered by exosomes in CC may modulate specific functions of other cells from the producer cells by adjusting related miRNA and regulate the progress of CC. However, related experiments are rare and relevant further studies are needed.

## Conclusion

Cervical cancer has remained a significant concern to women’s health so far. The unprecedentedly high incidence rate necessitates more attention and inquiry on the part of researchers. CircRNAs seem to serve a unique function in the course of CC: as a biomarker for CC, they may enhance the diagnostic efficiency or prognosis of CC. Additionally, Zhang et al. discovered that overactivation of the YAP pathway in CC induced by increased TFCP2 expression results in the formation and progression of CC, which is associated with the overexpression of hsa_ circ_ 0023404 (12). Naturally, the discovery process will raise some questions: Will circRNAs be regulated by several other miRNAs to influence downstream genes? Does crosstalk influence the expression of circRNAs in the etiology of CC? Future studies will provide light on the nature of these problems. At present, it is unclear whether manipulating circRNAs are advantageous for CC intervention. Thus, in addition to mechanistic study, more emphasis should be placed on the clinical value of circRNAs in CC. With the maturation of next-generation sequencing and microarray technologies, the mysteries of circadian regulation in CC and other illnesses will slowly be unveiled. In clinical therapy, specialists will finally grasp the most effective approach of manipulating circRNAs and applying it to the battle against sickness. We will continue to monitor this field in the future.

## Author Contributions

MW, YH, YuZ, QZ, GW, and CZ participated in the design of the review and drafted the manuscript. XG, KW, HF, BC, and HF collected the related literature and drafted the manuscript. MW, YH, XG, and KW revised and edited the manuscript. YL, YiZ, LZ, and GT proofread the language. MW, YH, and YuZ supervised the review process. All authors have read and approved the final manuscript.

## Conflict of Interest

The authors declare that the research was conducted in the absence of any commercial or financial relationships that could be construed as a potential conflict of interest.

## Publisher’s Note

All claims expressed in this article are solely those of the authors and do not necessarily represent those of their affiliated organizations, or those of the publisher, the editors and the reviewers. Any product that may be evaluated in this article, or claim that may be made by its manufacturer, is not guaranteed or endorsed by the publisher.

## Abbreviations

CC: cervical cancer
HPV: human papillomavirus
HIV: human immunodeficiency virus
AIDS: acquired immunodeficiency syndrome
ncRNAs: non-coding RNAs
miRNA: microRNA
snoRNA: small nucleolar RNA
lncRNA: long ncRNA
circRNA: circular RNA
HF: heart failure
PC: prostate cancer
ecircRNA: exonic circRNA
ciRNA: circular RNAs from introns
EIciRNA: exon–intron circRNA
NSCLC: non-small cell lung cancer
ESCC: esophageal squamous cell carcinoma
RBPs: RNA-binding proteins
COC: combination oral contraceptive
FIGO: the International Federation of Gynecology and Obstetrics
EMT: epithelial mesenchymal transition
CiRS-7: circular RNA sponge for miR-7
MREs: microRNA response elements
AGO: Argonaute
EIF3C: human Eukaryotic Translation Initiation Factor 3
YAP: Yes-associated protein
MAPK: mitogen-activated protein kinase
ERK1/2: extracellular signal-regulated kinase
PI3K: phosphatidylinositol 3-kinase
AUC: the area under the ROC curve
ROC: the receiver operating characteristics
TRAIL: tumor necrosis factor-related apoptosis-inducing ligand
EV: extracellular vesicles
HLECs: human lymphatic endothelial cells
GC: gastric cancer.
